# An Endowed Dental Dispensary for Rochester, New York

**Published:** 1915-08

**Authors:** 


### AN ENDOWED DENTAL DISPENSARY FOR ROCHESTER, NEW YORK.

George Eastman, president of the Kodak Company of Rochester, New York, has just announced to the Rochester Dental Society that he will erect a suitable building for a Dental Dispensary at an estimated cost of $300,000.00. Dr. Wm. W. Smith, who has for many years been greatly interested in the dental school children work, read the following announcement to the Dental Society, July 20th:

Through the generosity of George Eastman and the active interest of several prominent business men of this city, the Rochester Dental Society is about to realize an ambition which it has entertained for some years, and toward which it has been working steadily. Indeed, what has been done is far ahead of what any of the members of the Dental Society had dared to dream, for Rochester is to have a dental dispensary second to none in the country, and the only one of its kind except the Forsyth Dispensary in Boston.

The building will be three stories, of handsome design, and will cost about $300,000 fully equipped and including land. It will be provided with every convenience for advanced dental study and the carrying on of educational and research work. There will be about twenty chairs with competent operators at the start, and room for more as the work progresses. Provision will be made for performing all operations in oral surgery, all surgical and other treatments of the nose and throat when the condition of these organs is associated with oral diseases. Orthodontic work will be a special feature, as it has come to be known that narrow and contracted jaws and irregular teeth are a serious handicap to the child. The correction of these abnormal conditions is very important.

### Laboratory for Research.

A fine research laboratory is to be provided, where special attention will be given for studying the causes and effects of diseases of the mouth and teeth. There will also be a radiographic laboratory for X-ray work, which work is becoming very important in diagnosing dental diseases, and provision will be made for the extraction of teeth by the use of anesthetics. A very complete and perfect system for sterilizing all dental instruments will also be installed.

A fine lecture room, constructed with special attention to its acoustic properties and an amusement room will be attractive features of the structure. Here the children will be entertained and under the guise of amusement they will be taught the necessity for cleanliness in the care of their teeth. There will be regular courses of lectures for the internes and members of the staff, and a training school will be established for women who are preparing to take up prophylactic work. In time, it is planned to send these women into the schools of the city to do this work and to instruct the children as to the necessity for taking care of the teeth, thus opening up a new field for young women. This institution will be an ideal place in which they may qualify for this service. In some of the Eastern cities this school work has become an important part of preventive philanthropy, and there are places where no contagious diseases have been communicated among the children who are under proper dental care.

### Site in Main Street East.

The building will be erected in Main street east, near the corner of Alexander street, on the vacant lot between the chapter house of the Theta Delta Chi fraternity and the residence of Edward McSweeney. It will also have an opening on Kenilworth terrace, made possible by the purchase of a house and lot at the northwest corner of the street. Plans for the buildings have been prepared by Gordon & Madden, and it will be the most complete of its kind in the world.

For several months Mr. Eastman has been deeply interested in the subject of preventive dental work among children. He went as far as to visit the Forsyth Dispensary in Boston, one of the greatest institutions of its kind, and carefully studied its work, and the more he understood the scope of the work the more deeply interested he became. Then, after conference with William Bausch, who has always taken the keenest interest in this work and a committee from the Rochester Dental Society, he made the proposition to the society that he would build and equip a dental dispensary at a cost of from $250,000 to $300,000, this conditioned upon the willingness of the city to furnish at least $20,000 a year for five years, an amount sufficient to carry on the prophylactic work in the schools; that private citizens contribute $10,000 a year for five years, and Mr. Eastman himself would contribute $30,000 a year for five years. At the end of that time Mr. Eastman will furnish an endowment of $750,000 if these conditions have been met and the work is being carried on satisfactorily. He will then have paid into this enterprise over one million dollars.

### Superintendent to be Engaged.

The work has been thoroughly outlined and every detail has been gone into with Mr. Eastman. A superintendent will be engaged very soon, and a search is already on for a man who will be perfectly competent from a professional standpoint and big enough as an executive to fill this position. The work will be under his supervision from the very start, much the same as the work of the public libraries was developed under a newly appointed superintendent.

The work of the Rochester Dental Society looking toward a dispensary has been intelligent and persistent. The necessity for a place to take care of the teeth of the worthy poor of the city, especially the children, was early recognized, and the first institution of its kind was established at the Rochester City Hospital by members of this society some twenty-eight years ago and continued for two years, then abandoned because of lack of support.

A few members, however, refused to be balked, and persisted in the feasibility of re-establishing such an institution. A committee was appointed, and, after serving several years, reported that Captain Lomb would advance a sum of money sufficient to equip a free dental dispensary, to be the property of the society. On receipt of this report the committee was empowered to secure accommodations with some of the hospitals, but none was able to offer quarters with a suitable light and other essentials. Through the courtesy of the Public Health Dispensary this dispensary was located in its rooms, the association furnishing rooms, heat and electricity free to the Dental Society. This dispensary has been in successful operation ever since.

### Chartered by State Board.

The sum of $600 was tendered the society and was deemed sufficient, but through donations from local merchants, dental manufacturers and dealers, an equipment valued at $1,200 was secured free from all indebtedness. A charter was obtained from the New York State Board of Charities, and the dispensary opened to the public on Washington's birthday, February 22, 1905. It was kept open two afternoons each week, and twenty-four members alternated in attendance the first year. Captain Lomb then offered to pay the salary of one or more dentists who would be present each week day from 2 to 5 o'clock.

The Rochester Dental Society has been a pioneer in this work in this country. The first dental dispensary in any school in the United States was established in No. 14 School, in this city. Since that time the work has been taken up in the schools of many other cities, appropriating varying amounts from public funds for maintenance. Detroit has appropriated this year the sum of $30,000. The splendid gift of William Bausch made possible a complete equipment for No. 26 School, and recently Albert E. May offered to establish a dispensary in No. 18. Other public-spirited citizens have made like offers, especially in the case of No. 9, but these offers have not been considered, owing to the completion of the present plans. It has been decided that more efficient work can be done in this central dispensary, but trained workers will be sent to the schools with portable outfits for doing hygienic work, who will train the children in the care of the mouth and teeth.

### Encouraging to Society.

It will be remembered by many that when the actor William Hodge was in this city some years ago, he offered to give $1,000 to the charity which a committee should select as the most worthy. The committee reported in favor of the Dental Dispensary. This was another gift which helped to encourage those who were looking forward to the larger development of the work.

The dedication of the Forsyth Dental Infirmary, the work of which so impressed Mr. Eastman, took place last November and marked an epoch in dental history. At that time Thomas Alexander Forsyth said:

"It has been my wish that the Infirmary should be as a home to the children, beautiful and cheerful; a protector of their health, a refuge in their pain. By making them healthier and happier, I hope it may make them grow to be better citizens of our beloved Boston.

If this is accomplished, as I believe it must be, with the co-operation of the dental profession, I shall feel that the gift has been well bestowed.''

This is the idea that Mr. Eastman has in mind when the makes this princely gift to the children of Rochester, to the children who, for one reason or another, can not meet the expense of expert work, many of whom suffer all through life because of neglect of their teeth.

### Boston Dispensary Highly Prized.

The Forsyth building is partly of marble, and has been beautified especially since it is a memorial to James Bennett and George Henry Forsyth, brothers of the two Forsyths who erected it. There are many in the city of Boston who are of the opinion that never in the history of the world has money been so wisely expended as in this philanthropy. It has proved an uplift to the dental profession and will advance its power for efficient service.

The Rochester Dental Dispensary will serve the same purpose, and will prove one of the most far-reaching philanthropies ever advanced in this city. The number of diseases which may be traced to the teeth is increasing. The whole life of a person may be wrecked by neglect of the teeth in early youth. We are coming to understand this truth more perfectly and Rochester is taking its place as a pioneer in the advanced work of prevention which comes from a better understanding of the teeth and mouth.

The names of the trustees interested in this enterprise will be published later.

Resolutions thanking Mr. Eastman and Mr. Bausch were adopted by the society. They follow:

Resolved, That the Rochester Dental Society, pending further and more formal action, at a regular meeting, express to Mr. Eastman its appreciation of the exceptional opportunity afforded the society to extent and broaden its work of dental relief; and that it pledge him in this great benefaction its unqualified professional and practical support.

Resolved, That the thanks of the Rochester Dental Society are due and are herein extended to Mr. Bausch for the great interest he has taken in the work of the society; and also for the effective manner in which he has added to the possibilities of that work in meeting the conditions involved in Mr. Eastman's plans for the Rochester Dental Dispensary.

Dr. Rudolph Hofheinz was named by the society as its first representative on the Board of Trustees of the Dental Dispensary. Neither the number nor the names of the other members will be announced immediately. It is understood, however, that at least nine more will be named and that they will all be business men.

It was announced unofficially that the $750,000 endowment promised by Mr. Eastman is the first endowment made in his long list of benefactions in Rochester.

